# Knowledge hiding and individual task performance: The role of individual creativity as mediator

**DOI:** 10.1016/j.heliyon.2023.e21035

**Published:** 2023-10-21

**Authors:** Ika Atma Kurniawanti, Djumilah Zain, Armanu Thoyib, Mintarti Rahayu

**Affiliations:** a Department of Accounting, Faculty of Economics and Business, Airlangga University, Surabaya, Indonesia; b Doctoral Program of Management Science, Faculty of Economics and Business, Brawijaya University, Malang, Indonesia

**Keywords:** Knowledge hiding, Evasive hiding, Playing dumb, Rationalized hiding, Individual creativity, Individual task performance

## Abstract

This study investigates the association of knowledge hiding with individual task performance and the mediation role of individual creativity. It focuses on how employees' knowledge-hiding behavior can influence their task performance and how individual creativity can alleviate the negative consequences of knowledge-hiding. Data was collected from 256 employees working in financing companies in Indonesia. Hypothesis testing was conducted using the Structural Equation Model - Partial Least Square (SEM-PLS) model. The findings showed that all indicators significantly represented the dimensions of evasive hiding, playing dumb, and rationalized hiding, which collectively constitutes knowledge hiding. Furthermore, the study confirmed that individual creativity served as a mediator in the relationship between knowledge hiding and individual task performance. The implications of this study underscore the importance of addressing knowledge-hiding behavior within organizations and promoting individual creativity as a means to overcome the negative consequences and enhance task performance.

## 1 Introduction

Currently, organizations rely heavily on the knowledge and creativity of their employees to gain a competitive edge. However, a rising concern is the prevalence of knowledge-hiding behavior among employees, wherein individuals intentionally withhold or conceal valuable knowledge from others. This behavior can have detrimental effects on individuals and organizations, hindering collaboration, innovation, and overall performance. Knowledge management is a prominent topic in the field of organizational behavior and modern management, drawing the attention of scholars and practitioners alike. Knowledge management, which is based on the Knowledge-Based View (KBV) and Resource-Based View (RBV), recognizes knowledge as an intangible asset that not only enables organizations to sustain their long-term existence but also serves as a resource for competitive advantage [1,2]; Grant, 1996; [[3], [4], [5], [6], [7]]. KBV considers organizations as heterogeneous entities rich in knowledge that competitors find difficult to duplicate, laying the basis for sustainable differentiation and competitiveness (Leidner, 2015).

Knowledge hiding is a dyadic process involving two parties, namely the knowledge seekers and the knowledge providers [8]; Černe et al., 2019; [9,10]. Reciprocal effects in these dyadic interactions cannot be disregarded. Knowledge hiding is widely recognized as a counterproductive workplace behavior that impedes employees' creativity and degrades performance. Despite organizational efforts to encourage knowledge sharing, employees tend to hide their knowledge, both tacitly and explicitly [11]. When knowledge hiding occurs, the knowledge seeker does not obtain the requested knowledge or receives it in an unsatisfactory form, such as incomplete, delayed, or requiring additional processing. This disappointment may linger in subsequent interactions, fostering a vicious cycle of greater knowledge hiding among victims of such behavior. Empirical research indicates that negative behaviors have a greater and longer-lasting negative impact, exacerbating the perpetuation of knowledge hiding [12]; Xiong et al., 2017).

To gain a comprehensive understanding of knowledge dynamics in organizations, it is critical to give attention to knowledge hiding alongside knowledge sharing. Extensive empirical studies have consistently demonstrated the detrimental impact of knowledge hiding on organizational outcomes [[13], [14], [15]]; J [[16], [17], [18]]. Knowledge hiding disrupts the free flow of knowledge and collaboration among employees. A study revealed that the failure of knowledge-sharing initiatives resulted in annual losses of at least $31.5 billion for Fortune 500 companies [15]. Despite the increasing recognition of knowledge hiding as a significant phenomenon, there has not been a lot of research performed on the subject [8,10,13,17]. The field of knowledge management predominantly focuses on knowledge sharing, leaving knowledge hiding as a relatively novel concept.

Meanwhile [19,20], revealed different findings regarding the relationship between knowledge hiding and individual job performance. Their study reveals a positive association between knowledge hiding and individual job performance, which is mediated by well-being. However, the specific dimensions of knowledge hiding display varying relationships with individual job performance. Evasive hiding is positively related to innovative job performance but negatively associated with in-role job performance. On the other hand, playing dumb is only positively linked to in-role job performance, while rationalized hiding is solely positively related to innovative job performance. These discrepancies in results can be attributed to the researchers' distinct perspectives on the relationship between knowledge hiding and individual performance [21]. adopts a long-term perspective, while [20] focus on the short-term effects.

Consequently, current research primarily aims to explore the relationship between knowledge hiding and its antecedents and consequences, as well as investigate various organizational factors that may mediate or moderate this relationship. Given the nascent nature of knowledge hiding as a concept, there is ample room for research to delve into various aspects that influence its consequences and are affected by its antecedents. Furthermore, examining the mediating and moderating variables that underlie the relationship between knowledge hiding and its antecedents and consequences remains highly relevant in expanding our understanding of this phenomenon [22,23]. Therefore, researching these unexplored dimensions will contribute significantly to the knowledge management literature. The inconsistent findings between these studies highlight the existence of research gaps in understanding the relationship between knowledge-hiding and individual task performance. To determine whether individual task performance can be considered a consequence of knowledge-hiding, it is crucial to conduct further research into the specific association between knowledge-hiding and individual task performance. As a result, researchers can bridge the gaps in knowledge and provide a more comprehensive understanding of the impact of knowledge-hiding on individual performance outcomes.

Individuals within organizations rely on obtaining the necessary knowledge to support the creative process. However, when knowledge is deliberately withheld or hidden by colleagues, it hampers individual creativity. Empirical evidence consistently demonstrates the negative impact of knowledge-hiding on creativity. Several studies have revealed that knowledge-hiding directly decreases employee creativity [24] and team creativity, both directly and indirectly [25]. Similarly [13], found a negative relationship between knowledge-hiding and creativity. Notably, these studies focused on creativity at the organizational and team levels and the lack of research on the relationship between knowledge hiding and individual creativity. To address this research gap, examining the relationship between knowledge hiding and individual creativity is thus critical. Individual performance is dependent on the successful execution of the creative process, which involves generating, accumulating, and applying novel ideas, methods, and improvements to operational activities within the organization. When the individual creative process or individual creativity is impeded, it disrupts individual performance [26]. found that creativity has both a direct and indirect positive effect on individual task performance, further supporting the significance of creativity in individual performance. Additionally [27], affirmed the positive association between creativity and individual performance, providing consistent evidence of the relationship between these variables. Investigating the relationship between knowledge hiding and individual creativity will contribute to a more comprehensive understanding of how knowledge management practices impact individual performance outcomes.

This study aims to investigate the impact of knowledge hiding on individual task performance, with a specific focus on the mediating role of individual creativity. The objective is to examine how knowledge-hiding behaviors exhibited by employees can influence their task performance and how individual creativity alleviates the adverse effects of knowledge-hiding. We posit that knowledge-hiding behavior can detrimentally affect individual task performance through various mechanisms. Firstly, knowledge-hiding restricts access to critical information, impeding individuals' ability to acquire the necessary knowledge and skills to perform their tasks effectively [8,28]. Additionally, it hampers problem-solving capabilities as concealed knowledge deprives individuals of valuable insights and perspectives. Furthermore, knowledge-hiding stifles creativity by discouraging the exchange of ideas and inhibiting the exploration of innovative solutions. Conversely, we propose that individual creativity can serve as a mediator in the relationship between knowledge-hiding and task performance. By fostering the generation of novel ideas and promoting innovative thinking, individual creativity can counteract the negative consequences of knowledge-hiding. It empowers individuals to develop alternative approaches, overcome obstacles, and enhance their problem-solving abilities.

This study focuses on a sample of employees working within Indonesian financial firms. In the context of Indonesian workplace culture, the strong emphasis on maintaining harmonious relationships may contribute to the prevalence of knowledge hiding. However, the specific impact of such behavior on task performance remains unclear. Furthermore, the financial sector in Indonesia is characterized by strict legal and regulatory requirements, potentially motivating employees to conceal knowledge to avoid legal repercussions. This concealment could impede effective decision-making and process efficiency. Despite the recognition of the adverse consequences associated with knowledge hiding, there has been a noticeable dearth of research investigating its specific implications within Indonesia's financial landscape [29,30].

This study uniqueness lies on its contextual specificity, as it takes into account the distinctive cultural and practical facets of the Indonesian financial industry. By addressing this research gap, our study aimed to significantly enhance our comprehension of knowledge hiding and its influence on individual task performance, focusing on a sector that holds particular significance within the Indonesian economy. Therefore, this study aimed to address the following research questions (RQs).

RQ1 What is the relationship between knowledge hiding and individual task performance?

RQ2 Does individual creativity confirm to be a mediator in the relationship between knowledge hiding and individual task performance?

The anticipated outcomes of this study were poised to offer practical insights to managers, shedding light on strategies to mitigate the detrimental effects of knowledge hiding on individual task performance while considering the mediating role of individual creativity. This research was not only deemed necessary but also highly pertinent, as it delved into the intricacies of knowledge hiding and its repercussions on task performance within Indonesian financial companies. Moreover, the findings generated by this study were expected to provide invaluable guidance to financial institutions. These insights would assist in fostering a workplace culture that encourages openness and knowledge sharing, ultimately promoting a more creative environment. Furthermore, the study's outcomes were anticipated to be of relevance to policymakers and regulators. They could employ these insights to develop practices aimed at bolstering transparency, compliance, and effective knowledge management within the financial sector, thereby contributing to the industry's growth and stability.

## 2 Conceptual framework

### 2.1 Conservation of Resources theory (COR)

According to the Conservation of Resources (COR) theory by Hobfoll and Stevan (1989), individuals are naturally inclined to safeguard their valuable assets when confronted with actual or perceived threats of resource depletion. In professional settings, employees exhibit a proactive stance in resource protection. When they encounter stress-inducing factors like cynicism, role-related stress, and job insecurity, they may resort to knowledge concealment as a strategic measure to secure their resources [[31], [32], [33]]. While prior research has extensively explored the core tenets of the COR theory, this study aims to expand the horizons of investigation by delving into additional factors that can act as precursors to knowledge-hiding behaviors. These factors encompass evasive hiding, playing dumb, and rationalized hiding, offering valuable insights into how employees respond to resource-related threats within the workplace. Moreover, building on the findings of [34]; it's important to note that knowledge hiding has been empirically observed to exert a detrimental influence on individual performance. By concealing valuable knowledge, employees inadvertently limit their potential for excellence in task execution and effective fulfillment of their responsibilities, thereby impeding both individual and organizational objectives.

In addition to its impact on performance and creative endeavors, knowledge hiding is also linked to heightened levels of interpersonal distrust [8]. The distinctive work culture in Indonesia, characterized by collectivism, respect for authority, and an emphasis on harmony, plays a pivotal role in shaping knowledge-hiding behaviors like evasive hiding, playing dumb, and rationalized hiding. Within this cultural context, employees may feel compelled to avoid conflicts or confrontations with superiors or colleagues, prompting them to engage in knowledge-hiding behaviors to maintain social harmony [30]. Furthermore, the hierarchical structure inherent in Indonesian workplaces may discourage employees from freely expressing their expertise, resulting in a tendency to feign ignorance or withhold knowledge. These knowledge-hiding behaviors bear significant implications for employee performance. When employees or team members resort to evasive hiding, playing dumb, or rationalized hiding, essential information and valuable insights are withheld, hampering teamwork, decision-making, and problem-solving. Consequently, employee performance may decline as tasks become more demanding, time-consuming, and less effective [35].

In summary, the COR theory underscores employees' proactive efforts to safeguard their valuable resources when confronted with resource-related threats in the workplace. This study contributes to the existing body of research by exploring additional factors such as evasive hiding, playing dumb, and rationalized hiding, shedding light on their role as potential precursors to knowledge-hiding behaviors. Understanding these behaviors within the context of the Indonesian workplace culture provides valuable insights into their impact on employee performance and underscores the importance of cultivating a culture that promotes openness and knowledge sharing to enhance overall organizational effectiveness.

#### 2.1.1 Knowledge hiding constructs

The phenomenon of knowledge hiding, characterized by intentional withholding or concealment of requested knowledge by an individual, has far-reaching implications for organizations and interpersonal relationships within them [8]. Extensive research has illuminated various consequences of knowledge hiding, including detrimental effects on creativity [13,14,36], innovative work behaviors and individual performance [14]. Additionally, knowledge-hiding has been associated with the raised of interpersonal distrust [8] and strained relationships among individuals [37]. Remarkably, evidence suggests that knowledge concealment can spread rapidly, permeating from supervisors to subordinates (Arain, Bhatti et al., 2018).

The occurrence of knowledge-hiding is influenced by contextual factors. It tends to escalate in environments marked by growing distrust, declining competitiveness (Hernaus et al., 2018), or perceptions of organizational politics (Malik et al., 2019). Conversely, knowledge-hiding diminishes when reciprocal social exchange prevails, in climates fostering mastery [13], or when individuals possess a high level of evidential goal orientation or pro-social motivation [10,36]. However, the inconsistent findings between [19,20] stem from differing temporal perspectives adopted by the researchers when examining the relationship between knowledge-hiding and individual performance. While [19] adopted a long-term perspective [20], employed a short-term lens. Inconsistencies like these indicate existing research gaps. Therefore, further investigation is imperative to address whether individual task performance can be considered a consequential variable of knowledge-hiding.

Knowledge-hiding is a complex construct that encompasses three distinct dimensions, namely rationalized hiding, evasive hiding, and playing dumb. Rationalized hiding is the least deceptive dimension, characterized by explaining not sharing the knowledge. Evasive hiding involves misleading the requester by offering false or incomplete information or a promise of providing a more complete answer in the future. Playing dumb occurs when the hider feigns ignorance to avoid providing any information. Depending on the research objectives, knowledge-hiding can be assessed in different ways. If a theory focuses on a specific aspect of hidden knowledge, that facet can be studied individually. Alternatively, if the theory suggests potential interactions between the dimensions, they can be examined about one another. In cases where the entire construct is of interest, the complete measure should be used, with the items averaged. Given the deceptive nature of knowledge-hiding, a self-report measure is suitable, as others may underestimate or overestimate the frequency of knowledge being hidden from them.

Empirical studies have explored knowledge-hiding from psychological and personality perspectives, highlighting various factors and their effects [[38], [39], [40]]; Arain, Hameed et al., 2020 [8,28,41]; Hameed, 2012 [12,20,42]; Xiong et al., 2017). To measure the dimensions of knowledge-hiding, the model developed by Ref. [8] is utilized, employing a modified questionnaire with a 7-point Likert scale. Confirmatory factor analysis is employed to test the indicators and dimensions of knowledge-hiding using a second-order approach. In this study, the following hypotheses are proposed.

H1a The indicators in the evasive hiding dimension manifest/reflect the knowledge-hiding construct.

H1b The indicators in the playing dumb dimension manifest/reflect the knowledge-hiding construct.

H1c The indicators in the rationalized hiding dimension manifest/reflect the knowledge-hiding construct.

#### 2.1.2 The association of knowledge hiding on individual task performance

Knowledge-hiding behavior within an organization leads to incomplete, inappropriate, and insufficient information being shared among its members. This situation hampers the smooth execution of individual tasks, thereby affecting task performance. Moreover, knowledge hiders may also suffer from a knowledge deficit in the future, further impacting their task performance. Although [19] is the sole researcher who has explored the relationship between knowledge-hiding and task performance, his findings conclusively demonstrate that knowledge-hiding has a detrimental effect on task performance. By empirically investigating the impact of knowledge-hiding on task performance, this study added to the existing literature, providing additional evidence to support the negative relationship between knowledge hiding and task performance. The findings will assist organizations in recognizing the significance of addressing knowledge-hiding behaviors and developing strategies to foster a more transparent and collaborative work environment, ultimately enhancing overall task performance. To validate and reinforce [19] results, this study aims to test the following hypothesis.

H2 Knowledge-hiding has a negative association with individual task performance.

#### 2.1.3 The association of knowledge hiding on individual creativity

The generation of creative ideas heavily relies on the exchange of perspectives and approaches through social interactions [13]. further suggested that individuals who engage in extensive knowledge concealment experience a decrease in their creativity levels. It is argued that knowledge sharing fosters creativity [41], and when employees choose to hide their knowledge, it disrupts the reciprocal process in which colleagues feel encouraged to share their ideas, knowledge, and information. Existing literature consistently demonstrates a negative relationship between knowledge concealment and employee creativity [13,41]. Based on this argument, it is possible to conclude that knowledge hiding among employees in an organization has a detrimental impact on individual creativity, subsequently affecting individual, team, and organizational performance. Consequently, this diminishes the collaborative environment within the organization, leading to poor overall performance and potentially jeopardizing the organization's success [42]. Although there is a lack of empirical investigation into the direct effect of knowledge-hiding on individual creativity, earlier studies have shown that knowledge-hiding negatively affects creativity at the group level [25] and at the organizational level [13,24]. Considering the findings from these studies, it is reasonable to hypothesize that knowledge-hiding also influences creativity at the individual level. Therefore, the following hypothesis is formulated.

H3 Knowledge-hiding has a negative association on individual creativity.

#### 2.1.4 The association of individual creativity on individual task performance

Individual creativity has a significant impact on the effectiveness and efficiency of individual performance within an organizational setting. Individuals who can tap into their creative potential are more likely to approach tasks and assignments with innovative thinking and problem-solving strategies. This ability to generate novel and valuable ideas empowers individuals to devise unique approaches and solutions, ultimately leading to enhanced task performance. Empirical evidence supports the hypothesis that individual creativity has a positive impact on task performance [26]. conducted a study that demonstrated a direct and indirect relationship between creativity and individual performance. The findings revealed that individuals who exhibit higher levels of creativity not only demonstrate better task performance but also indirectly influence their performance through various mediating factors, such as motivation and job satisfaction. These results highlight the vital role of individual creativity in driving superior performance outcomes.

Furthermore [27], performed research that further strengthens the argument for a positive association between creativity and individual performance. Their findings demonstrated a positive relationship, indicating that individuals who possess greater creative abilities also tend to exhibit higher levels of performance. This suggests that creativity enhances individuals' capacity to excel in their tasks and deliver exceptional outcomes. In light of the existing empirical support, it is reasonable to hypothesize that individual creativity exerts a positive effect on task performance. When individuals can harness their creative potential, they are better equipped to tackle challenges, think outside the box, and find innovative solutions. As a result, their task performance should be more effective, efficient, and impactful. Therefore, it is reasonable to hypothesize that individual creativity exerts a positive effect on task performance, which serves as a proxy for measuring individual performance. Based on this rationale, the following research hypothesis is proposed.

H4 Individual creativity has a positive association on individual task performance.

#### 2.1.5 The role of individual creativity as a mediating variable

Knowledge-hiding refers to the deliberate act of withholding or concealing information that is requested by others [8]. It has been empirically established that knowledge-hiding has negative consequences not only on task performance but also on creativity [19]. conducted a study that specifically examined the impact of knowledge-hiding on task performance. The results indicated a negative relationship. Individuals who engage in knowledge-hiding behaviors reduce the availability of crucial information required to perform tasks effectively, leading to a decline in task performance. Individuals who withhold or hide knowledge impede their own and others' ability to obtain and use relevant information, limiting problem-solving, decision-making, and overall task execution. Moreover, previous research has consistently demonstrated a negative association between knowledge hiding and creativity [13,14,25]. conducted studies that revealed a detrimental effect of knowledge hiding on creativity. When individuals intentionally conceal knowledge, it restricts the flow of information and inhibits the exchange of ideas and perspectives that are essential for generating creative insights. Consequently, knowledge hiding stifles the creative process and reduces individuals' ability to generate innovative solutions and approaches.

On the other hand, creativity has been shown to have a positive influence on performance [26]. demonstrated that creativity directly and indirectly affects individual performance. Creative individuals possess the capacity to think divergently, envision alternative possibilities, and devise unconventional strategies to overcome challenges. This innovative thinking and problem-solving ability enhances task performance by enabling individuals to approach their work with fresh perspectives and novel solutions. Similarly [27], found a positive relationship between creativity and individual performance. The study revealed that individuals who exhibit higher levels of creativity tend to demonstrate superior performance outcomes. The creative process not only fosters higher quality work but also increases individuals' motivation, engagement, and overall job satisfaction, leading to enhanced performance levels. Based on the existing empirical evidence, it is reasonable to propose that knowledge-hiding has an indirect effect on task performance through its negative impact on individual creativity. When individuals engage in knowledge-hiding behaviors, it restricts access to essential knowledge and information needed to support the generation of creative ideas. Consequently, this hampers the development of innovative approaches and limits individuals' ability to perform tasks optimally. Therefore, it is reasonable to suspect that knowledge hiding has an indirect effect on individual performance through individual creativity. Based on this rationale, the proposed hypothesis is.

H5 Individual creativity mediates the association between knowledge hiding and individual task performance.

## 3 Research methodology

**Fig. 1:**
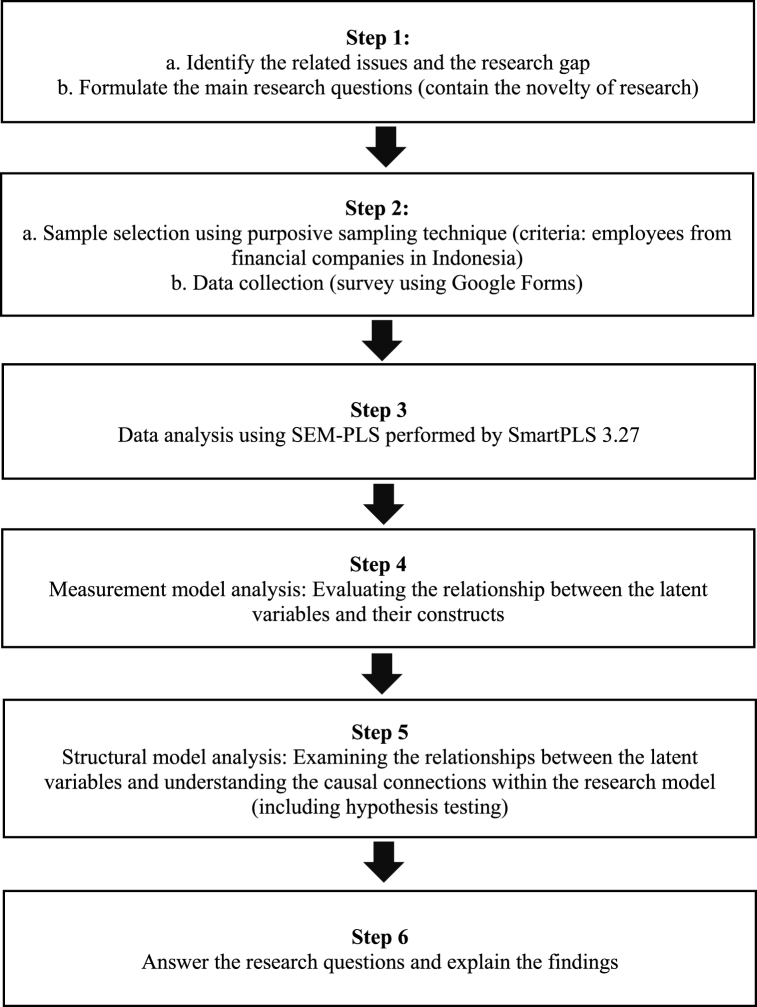
Research design.

Source: Authors elaboration, 2023.

**Fig. 2:**
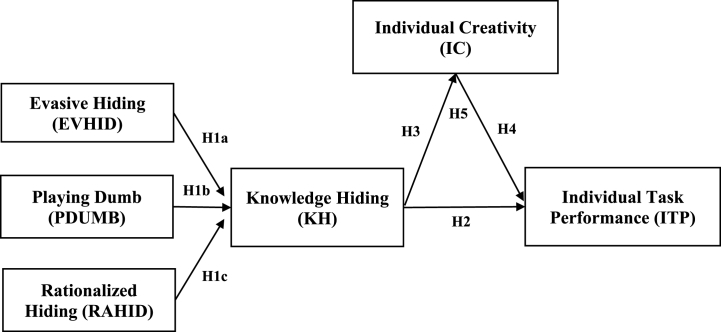
Conceptual framework.

Source: Authors elaboration, 2023.

This study employed a quantitative research approach to investigate the relationship between knowledge hiding, individual creativity, and task performance (Fig. 2). This study utilized a purposive sampling technique, a deliberate method of sample selection guided by predefined criteria set by the authors. This approach facilitated the careful selection of participants possessing characteristics or qualities pertinent to the research objectives, offering valuable insights into the subject under investigation. The utilization of purposive sampling ensured that the chosen participants met the desired criteria, enhancing the pertinence and applicability of the study's findings. Fig. 1 provides an overview of the research design, encompassing problem identification, formulation of research questions, the purposive selection of employees from financial firms in Indonesia, data collection through a survey, SEM-PLS analysis, examination of latent variable relationships, hypothesis testing, and the presentation of insights addressing the research questions.

The study involved 256 employees from Indonesian financial firms, specifically selected due to their direct involvement in pertinent organizational processes. This selection was driven by the intricacies of the financial industry and the significance of knowledge sharing and task performance. Furthermore, this research aimed to investigate knowledge hiding and its implications for individual task performance within the context of financial company employees. Additionally, it sought to explore the mediating role of individual creativity. Conducting research in Indonesian financial firms is notable due to the distinct cultural, organizational, legal, and practical aspects of this industry [29,30]. By addressing this research gap, the study intended to offer valuable insights into the impact of knowledge hiding on task performance within a pivotal sector of the Indonesian economy. Notably, the emphasis on interpersonal harmony and the preservation of positive relationships in Indonesian culture may lead to hesitancy in knowledge disclosure, potentially resulting in prevalent knowledge hiding in the workplace. Furthermore, stringent legal and regulatory requirements within the financial sector may incentivize employees to conceal knowledge to avoid legal repercussions or breaches, which could affect individual task performance.

Initially, questionnaires were distributed to 300 potential respondents. To enhance research credibility and mitigate biases, purposive sampling was employed, ensuring the inclusion of participants possessing specific qualities aligned with the research objectives. By targeting employees directly engaged in critical organizational processes, the sample closely represented the intended population of Indonesian financial firms, reducing the risk of selection bias.

The authors employed a combination of direct distribution and an online survey using Google Forms to reach a diverse group of potential respondents, mitigating potential biases associated with relying solely on one data collection method. Additionally, to enhance the study's quality and minimize biases, a pilot test was conducted on 30 respondents using the SEM-PLS model [43]. This pilot test evaluated the validity of the instruments and the reliability of the measurement scales utilized in the study. It also assessed the clarity of survey questions and instructions, aiming to gather valuable feedback from respondents to address any ambiguities or confusion. The survey questions were designed based on the operationalization of the research variables, seeking to capture participants' perceptions regarding knowledge hiding, individual task performance, and the mediating role of individual creativity. The questionnaire comprised a total of 21 questions, each addressing specific aspects of the variables examined (refer to Table 1).

**Table 1:** Question statements.

Variables	Code	Question statements
Knowledge hiding (KH)		
Evasive hiding (EVHID)	EVHID1	I agree to help even if it's out of compulsion
	EVHID2	I agree to help but will provide information that does not exactly match the information requested
	EVHID3	I told them I would help later, but I would hold off on helping as long as I could
	EVHID4	I will try to offer other information, but different from what was requested
Playing dumb (PDUMB)	PDUMB1	I pretended not to know the requested information
	PDUMB2	I would say I don not know about the information requested, but the fact is I do
	PDUMB3	I pretended not to know about the information being discussed
	PDUMB4	I confess that I am not very familiar with the topic or information
Rational hiding (RAHID)	RAHID1	I explained that I actually wanted to share the information, but I was not allowed to
	RAHID2	I explained that the information is confidential and can only be known by people involved in certain projects.
	RAHID3	I informed that my supervisor does not allow anyone to share this information
	RAHID4	I declare that I will not answer related questions
**Individual creativity (IC)**		
Individual creativity	IC1	I have the opportunity to use my creative skills and abilities at work
	IC2	I was invited to submit ideas for improvement and development in the workplace
	IC3	I have the opportunity to participate in a team
	IC4	I have freedom to decide how I will complete my work tasks
	IC5	My creative abilities are fully utilized to develop my potential
**Individual task performance (ITP)**		
Individual task performance	ITP1	Individual employees always plan their work to be completed on time
	ITP2	Individual employees determine the targets or results to be achieved in their work
	ITP3	Individual employees are able to separate the main problems and side problems at work
	ITP4	Individual employees are able to do a good job with optimal time and effort

The collected data were subjected to rigorous statistical analysis using SPSS 25. Descriptive statistics were employed to examine the demographic characteristics of the sample and provide an overview of the participant's responses. This analysis allowed for a comprehensive understanding of the participants' profiles, such as their age, gender, education level, and work experience. To test the research hypotheses, a Structural Equation Model - Partial Least Square (SEM-PLS) analysis was conducted using the SmartPLS 3.27 software. SEM-PLS is a widely used statistical technique that enables the assessment of complex relationships among latent variables. It is particularly suited for studies with smaller sample sizes and exploratory research objectives. This study utilized SEM-PLS to assess the direct and indirect effects of knowledge hiding on task performance through individual creativity.

SEM-PLS (Structural Equation Modeling-Partial Least Squares) model, pioneered by Herman OA Wold, has gained significant traction in various social science disciplines, including organizational and business management (Hair et al., 2012). This modeling approach offers researchers the capability to estimate intricate models comprising multiple constructs, indicator variables, and structural path models, all without imposing stringent assumptions of normal distribution [45]. Its flexibility makes it particularly suitable for research endeavors that possess weak theoretical foundations [46]. SEM-PLS analysis encompasses two essential stages: the measurement model analysis and the structural model analysis. In the measurement model analysis, several key components are evaluated, including loading factor values, average variance extracted (AVE), and reliability tests such as Cronbach's alpha. Convergent validity is deemed adequate when the loading factor and AVE values surpass the threshold of 0.7 [43]. Reliability is considered satisfactory when Cronbach's alpha exceeds 0.7. This stage allows researchers to assess the measurement properties of the constructs employed in the model. The subsequent stage entails the structural analysis, which delves into the relationships between the endogenous and exogenous variables, shedding light on the causal connections within the research model. By utilizing SEM-PLS analysis, this study aimed to contribute to the existing literature by uncovering the intricate dynamics between knowledge hiding, individual task performance, and the role of individual creativity as a potential moderator.

## 4 Findings

Table 2 provides an overview of the general characteristics of the participants involved in this study, encompassing a total of 256 respondents. The characteristics examined include gender, age, division/department, and tenure within the organization. Notably, the characteristics of the respondents indicate a prevailing male representation, accounting for 84.4% of the sample. In terms of age, 59.4% of respondents are between the ages of 36 and 45, representing a significant proportion. Additionally, the tenure of the respondents within their respective organizations is explored, offering insights into their length of service. It is worth noting that a considerable proportion of the participants (37.9%) have a work tenure of less than 5 years.

**Table 2:** Characteristics of respondents.

Characteristics of respondents	Frequency	Percentage
**Gender**		
Male	216	84.4
Female	40	15.6
**Total**	**256**	**100**
**Age**		
<25 years old	4	1.6
26–35 years old	75	29.3
36–45 years old	152	59.4
>45 years old	25	9.8
**Total**	**256**	**100**
**Devision**		
Collection	120	46.9
Operation	22	8.6
Marketing	32	12.5
HR & General Admin	27	10.5
Others	55	21.5
**Total**	**256**	**100**
**Tenure**		
<5 years	97	37.9
6–10 years	44	17.2
11–15 years	76	29.7
16–20 years	36	14.1
>20 years	3	1.2
**Total**	**256**	**100**

**Fig. 3:**
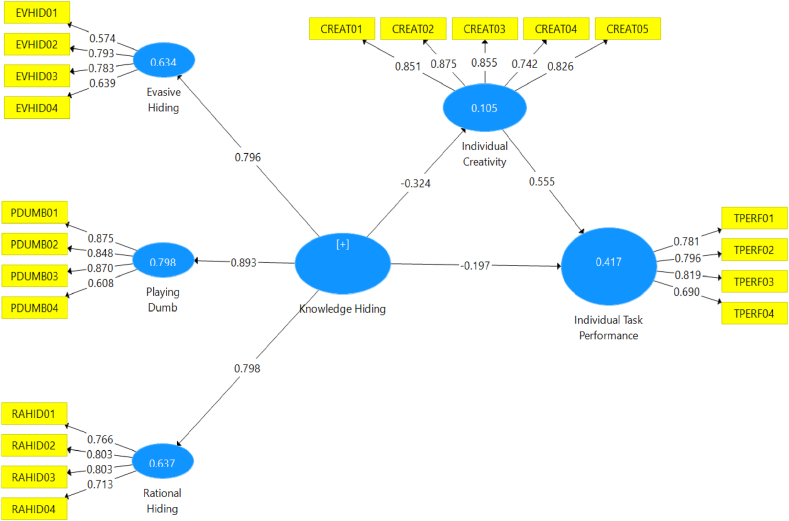
Results of the structural model estimation test using algorithm technique.

Source: Authors elaboration, 2023.

The analysis in this study consists of two main stages: the outer model testing phase and the inner model testing phase. The outer model testing phase assesses the validity and reliability of the indicators used to measure the construct, while the inner model testing phase examines the research hypotheses. The Structural Equation Model - Partial Least Squares (SEM PLS) was employed for the structural model testing. In this study, all hypotheses were tested simultaneously, as depicted in Fig. 3, using the algorithm technique method. During the measurement model testing phase, various assessments were conducted to ensure the reliability and validity of the PLS analysis. Convergent validity, discriminant validity, and composite reliability were examined to determine if the indicators in the PLS model satisfied the required criteria. These results are critical in evaluating the research hypothesis, as they indicate the extent to which the indicators accurately measure the construct under investigation.

To assess convergent validity, the loading factor values of the indicators to their respective constructs are examined. In confirmatory research, a loading factor above 0.7 is considered acceptable, while for exploratory research, a threshold of 0.6 is used. Table 3 displays the results of convergent validity test in the model. It is shown that all indicators demonstrate loading factors above 0.6, indicating convergent validity. Furthermore, the Average Variance Extracted (AVE) values of each construct are evaluated. If the AVE value for each construct exceeds 0.5, it indicates that convergent validity is satisfied. Table 3 also shows the complete AVE values for each construct, confirming their convergent validity.

**Table 3:** The results of SEM outer model.

Item	Cronbach's Alpha	Rho_A	Composite Reliability	Average Variance Extracted (AVE)
Evasive hiding (EVHID)	0.754	0.778	0.794	0.595
Playing dumb (PDUMB)	0.814	0.832	0.881	0.653
Rational hiding (RAHID)	0.773	0.771	0.855	0.596
Knowledge hiding (KH)	0.861	0.871	0.888	0.603
Individual creativity (IC)	0.888	0.895	0.918	0.691
Individual task performance (ITP)	0.774	0.778	0.855	0.598

To ensure that each concept within a latent variable is distinct from other variables, discriminant validity is also essential. It is assessed by examining the cross-loading factor of each indicator to its respective construct. If the cross-loading value of an indicator to its construct is higher than the cross-loading values to other constructs, it implies that the indicator meets the criterion for discriminant validity. This analysis helps establish the distinctiveness of each construct and ensures that they do not overlap with one another.

The cross-loading factors are shown in Table 4 and are used to assess the discriminant validity of the indicators. The cross-loading factors indicate the extent to which an indicator is associated with its construct compared to other constructs. To establish discriminant validity, the cross-loading value of an indicator on its construct should be higher than its cross-loading values on other constructs. By examining the cross-loading factors, it can be determined whether the indicators meet the criteria for discriminant validity, thereby ensuring that each construct is distinct from others and not overlapping.

**Table 4:** Cross loading factors.

	Evasive hiding	Playing dumb	Rational hiding	Individual creativity	Individual task performance
EVHID01	0.674	0.271	0.339	−0.195	−0.278
EVHID02	0.793	0.436	0.248	−0.113	−0.187
EVHID03	0.783	0.579	0.378	−0.264	−0.301
EVHID04	0.639	0.406	0.262	−0.101	−0.073
PDUMB01	0.564	0.875	0.457	−0.190	−0.284
PDUMB02	0.522	0.848	0.398	−0.197	−0.261
PDUMB03	0.526	0.870	0.466	−0.245	−0.255
PDUMB04	0.367	0.608	0.465	−0.187	−0.211
RAHID01	0.366	0.437	0.766	−0.232	−0.230
RAHID02	0.295	0.369	0.803	−0.260	−0.203
RAHID03	0.290	0.366	0.803	−0.228	−0.262
RAHID04	0.387	0.509	0.713	−0.238	−0.288
IC01	−0.251	−0.270	−0.299	0.851	0.539
IC02	−0.200	−0.211	−0.244	0.875	0.523
IC03	−0.219	−0.260	−0.271	0.855	0.561
IC04	−0.132	−0.142	−0.221	0.742	0.441
IC05	−0.203	−0.149	−0.250	0.826	0.494
ITP01	−0.263	−0.168	−0.165	0.524	0.781
ITP02	−0.224	−0.265	−0.215	0.449	0.796
ITP03	−0.282	−0.286	−0.352	0.487	0.819
ITP04	−0.159	−0.257	−0.254	0.447	0.690

In addition to the conventional methods of assessing discriminant validity, such as the Fornell-Larcker criterion and cross-loading examination, this research also employed an evaluation of the Heterotrait-Monotrait Ratio of Correlations (HTMT) values. The findings of the HTMT values are presented in Table 5. The HTMT table demonstrates values below 0.90, signifying the successful establishment of discriminant validity between two reflective constructs [47].

**Table 5:** Heterotrait-monotrait ratio (HTMT).

	Evasive Hiding	Individual Creativity	Individual Task Performance	Playing Dumb	Rational Hiding
Evasive Hiding					
Individual Creativity	0.314				
Individual Task Performance	0.418	0.744			
Playing Dumb	0.827	0.295	0.399		
Rational Hiding	0.614	0.369	0.407	0.703	

Following the rigorous evaluation of discriminant validity, our study proceeded to examine Collinearity Statistics, with a specific focus on the Variance Inflation Factor (VIF), as presented in Table 6. It is imperative to underscore that formative measurement models do not inherently yield high correlations among their constituent indicators, as these indicators fundamentally serve non-interchangeable roles. Notably, the emergence of substantial correlations between two indicators operating within a formative construct is unequivocally acknowledged as an instance of collinearity [48]. Excessive collinearity among formative indicators is deemed problematic due to its pronounced influence on the accurate estimation of indicator weights and the associated statistical significance. A VIF value reaching or surpassing the threshold of 5 serves as an unmistakable signal indicative of a potential issue related to collinearity [49]. To measure the extent of collinearity within the framework of Partial Least Squares Structural Equation Modeling (PLS-SEM), this study methodically conducted an assessment of the Variance Inflation Factor (VIF). The results of the collinearity examination confirm that all indicators boast VIF values residing below the 5-threshold, thereby affirming the complete absence of any observable collinearity concerns within the model.

**Table 6:** Collinearity statistics (VIF).

Variables	VIF
EVHID02	1.508
EVHID03	1.372
EVHID04	1.183
EVHID01	1.141
IC01	2.573
IC02	3.034
IC03	2.396
IC04	1.883
IC05	2.330
ITP01	1.563
ITP02	1.667
ITP03	1.691
ITP04	1.303
PDUMB01	2.519
PDUMB02	2.483
PDUMB03	2.205
PDUMB04	1.263
RAHID01	1.466
RAHID02	2.009
RAHID03	1.971
RAHID04	1.312

Moreover, the subsequent step involves analyzing the inner model. The inner model analysis comprises several tests, including the assessment of the significance of direct influence, the examination of indirect effects, and the measurement of the impact of each exogenous variable on the endogenous variable. These tests were conducted to evaluate the research hypotheses. Specifically, the significance test for direct effects was utilized to examine the influence of exogenous variables on endogenous variables. In the case of a one-way hypothesis, if the p-value is less than 0.05 and the t-statistic exceeds 1.96, the null hypothesis (H0) is rejected, indicating a significant effect of the exogenous variable on the endogenous variable.

**Fig. 4:**
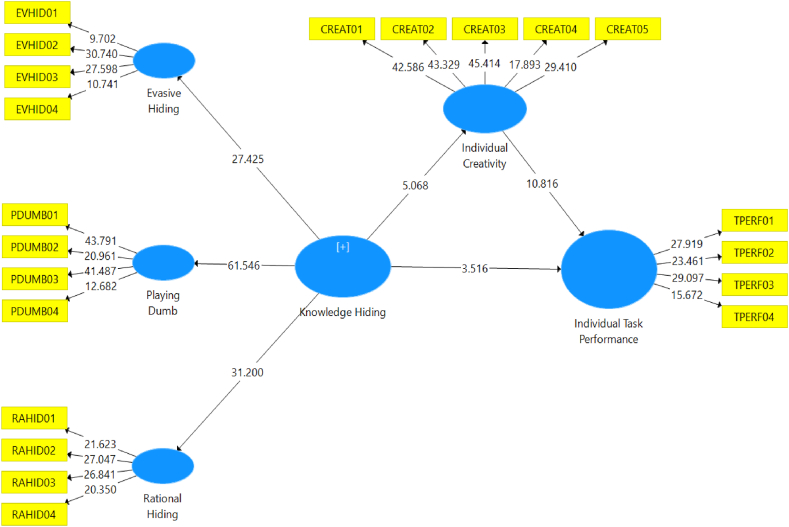
Structural model estimation testing using the bootstrapping technique.

Source: Authors elaboration, 2023.

Furthermore, the results of the significance test provide insights into the direction of the relationship between exogenous and endogenous variables. The original sample values for each relationship reveal the direction of the relationship. If the direction is positive, it indicates a positive unidirectional effect of the exogenous variable on the endogenous variable. Conversely, if the original sample value is negative, it suggests a negative inverse relationship between the exogenous variable and the endogenous variable. Fig. 4 presents the results of the model estimation conducted in this study, providing a visual representation of the relationships among the variables.

A thorough analysis was performed to examine the direct effect, indirect effect, and total effect in the research framework. The outcomes of these analyses are presented in Table 7, providing valuable insights into the hypotheses being tested. These findings enhance our understanding of the relationships and effects within the research framework, contributing to a comprehensive comprehension of the proposed hypotheses.

**Table 7:** The direct relationships of the structural model.

Hypotheses	O	Mean	STDEV	T-Statistics	P Values
KH - > EVHID	0.796	0.800	0.029	27.425	0.000
KH - > PDUMB	0.893	0.894	0.015	61.546	0.000
KH - > RAHID	0.798	0.798	0.026	31.200	0.000
KH - > ITP	−0.197	−0.195	0.056	3.516	0.000
KH - > IC	−0.324	−0.330	0.064	5.068	0.000
IC - > ITP	0.555	0.558	0.051	10.816	0.000

The analysis results indicate that evasive hiding, with a loading factor of 0.796 and a T-statistic of 27.425 (p-value = 0.000), manifests the knowledge-hiding construct. Similarly, playing dumb, with a loading factor of 0.893 and a T-statistic of 61,546 (p-value = 0.000), and rationalized hiding, with a loading factor of 0.798 and a T-statistic of 31.200 (p-value = 0.000), also manifest the knowledge hiding construct. These findings highlight that knowledge-hiding has a negative and significant effect on task performance, as evidenced by its loading factor of −0.197, T-statistic of 3.516, and p-value of 0.000. Similarly, knowledge-hiding also has a negative effect on individual creativity, as indicated by its loading factor of −0.324 and T-statistic of 5.068, although the effect is not statistically significant. On the other hand, individual creativity exhibits a positive and significant effect on individual task performance, with a loading factor of 0.555, a T-statistic of 10,816, and a p-value of 0.000. These findings add to our understanding of the relationships between knowledge-hiding, task performance, and individual creativity in the research context.

In this study, individual creativity is considered as a mediating variable that intervenes in the relationship between knowledge hiding and task performance. The significance level of 5 % is used to assess the hypothesis. If the p-value is less than 0.05, the null hypothesis (Ho) is rejected, indicating a significant indirect effect. Conversely, if the p-value is greater than 0.05, the null hypothesis (Ho) is accepted, suggesting no significant indirect effect. The findings of the indirect effect analysis can be found in Table 8, providing insights into the mediating role of individual creativity in the relationship between knowledge hiding and task performance.

**Table 8:** Indirect association test results.

Hypotheses	O	Mean	STDEV	T-Statistics	P Values
KH - > IC - > ITP	−0.180	−0.184	0.038	4.711	0.000

The analysis reveals that knowledge-hiding behavior has a notable adverse influence on individual creativity, and subsequently, individual creativity significantly negatively impacts individual task performance. These findings confirm the proposed mediating role of individual creativity in the relationship between knowledge hiding and individual task performance. The results indicate that individual creativity partially mediates the negative association between knowledge hiding and individual task performance. Hence, simply addressing knowledge-hiding behavior may not be sufficient to enhance individual task performance; fostering individual creativity is also crucial. The outcomes of the direct, indirect, and total effect tests are summarized in Table 9, providing a comprehensive overview of the hypotheses testing outcomes.

**Table 9:** Summary of hypothesis testing.

Hypotheses	Beta	STDEV	T-Statistics	P Values
H1a: KH - > EVHID	0.796	0.029	27.425	0.000
H1b: KH - > PDUMB	0.893	0.015	61.546	0.000
H1c: KH - > RAHID	0.798	0.026	31.200	0.000
H2: KH - > ITP	−0.197	0.056	3.516	0.000
H3: KH - > IC	−0.324	0.064	5.068	0.000
H4: IC - > ITP	0.555	0.051	10.816	0.000
H5: KH - > IC - > ITP	−0.180	0.038	4.711	0.000

Table 9 shows that evasive hiding, as indicated by a loading factor of 0.796 (T-statistic = 27.425, p-value = 0.000), significantly reflects the knowledge-hiding construct, supporting hypothesis 1a. Similarly, playing dumb, with a loading factor of 0.893 (T-statistic = 61.546, p-value = 0.000), significantly manifests the knowledge-hiding construct, supporting hypothesis 1 B. Additionally, rationalized hiding, with a loading factor of 0.798 (T-statistic = 31,200, p-value = 0.000), significantly reflects the knowledge hiding construct, supporting hypothesis 1. Furthermore, knowledge hiding exhibits a negative effect on task performance, with a loading factor of −0.197 (T-statistic = 3.516, p-value = 0.000), providing statistical support for hypothesis 2. Similarly, knowledge hiding demonstrates a negative association on individual creativity, with a loading factor of −0.324 (T-statistic = 5.068, p-value = 0.000), supporting hypothesis 5. Conversely, individual creativity has a positive and significant association with individual task performance, with a loading factor of 0.555 (T-statistic = 10,816, p-value = 0.000), confirming hypothesis 6. Importantly, knowledge hiding's negative association on task performance is mediated by individual creativity, with a loading factor of −0.180 (T-statistic = 4,711, p-value = 0.000). This finding supports hypothesis 7, highlighting that individual creativity significantly mediates the relationship between knowledge hiding and task performance.

## 5 Discussion

This study aims to examine and validate the indicators and dimensions of knowledge hiding using second-order confirmatory factor analysis. The findings of this study reveal that all indicators significantly manifest the dimensions of evasive hiding, playing dumb, and rationalized hiding. The findings of this study also indicate a negative association between knowledge hiding and task performance. It aligns with prior research, particularly the work of [19]; which highlights the adverse impact of knowledge hiding on task performance. Knowledge hiding can disrupt the flow of complete and relevant information within an organization, potentially leading to a knowledge deficit among those engaging in this behavior. Consequently, it can hinder their task performance. However, it's worth noting that [19] is one of the few studies explicitly examining this relationship, providing evidence of its negative association. Furthermore, knowledge hiding has been consistently associated with adverse consequences for interpersonal relationships within organizations, as evidenced by previous research [8]; Cerne et al., 2014; [17]. It also negatively associates with extra-role performance, as individuals practicing knowledge hiding may neglect non-formal roles that could otherwise strengthen relationships among organizational members.

Moreover, the findings of this study confirmed that knowledge hiding has a negative and statistically significant association on individual creativity. These results align with previous research that has consistently demonstrated the detrimental association of knowledge hiding on individual creativity. Knowledge hiding is viewed as a behavior that hinders individual creativity and innovation within organizations [28]. On the other hand, knowledge sharing has been found to have a positive and significant influence on individual creativity and innovation in companies. For individual creative processes to thrive within organizations, the knowledge necessary to support these processes must be readily accessible. When individuals engage in knowledge hiding by withholding or concealing information, the flow of necessary knowledge is impeded. Consequently, individual creativity is hindered and cannot reach its optimal potential. While empirical investigations specifically examining the impact of knowledge-hiding on individual creativity are scarce, existing studies have shown that knowledge-hiding negatively affects creativity at the group level (Fong et al., 2017) and at the organizational level (Cerne et al., 2014; [24]. Building upon the findings of these studies, it is reasonable to infer that knowledge-hiding also has a negative association with individual creativity. Overall, by highlighting the negative association of knowledge concealing with individual creativity, these findings add to the increasing body of research on this topic. By recognizing the adverse consequences of knowledge hiding on individual creative processes, organizations can better understand the value of fostering a culture of knowledge sharing and transparency to boost creativity and innovation.

Moreover, individual creativity has a positive and significant association with task performance, underscoring the importance of creativity in enhancing overall performance within organizations. The study also reveals that individual creativity acts as a mediator in the relationship between knowledge hiding and task performance. In essence, individual creativity serves as a bridge that translates the negative impact of knowledge hiding into reduced task performance. In summary, knowledge hiding presents complex trade-offs and implications within organizational contexts. While it may serve as a means to protect valuable knowledge resources and potentially maintain positive relationships, it can hinder both task performance and individual creativity. Recognizing the intricate interplay between these factors is vital for organizations seeking to optimize their performance outcomes and foster a culture of knowledge sharing and transparency to boost creativity and innovation.

This findings aligned with the conceptualization and measurement of knowledge hiding developed by Refs. [[31], [32], [33]]; and [8]; in which knowledge hiding is defined as the deliberate act of individuals to purposefully withhold or hide knowledge that has been requested by others. Specifically in the Indonesian context, the most prevalent dimensions of knowledge hiding are playing dumb, rationalized hiding, and evasive hiding. Pretending not to know is perceived as a safer behavior for knowledge hiding, as it avoids potential repercussions compared to engaging in rationalized hiding or evasive hiding. Previous empirical investigations, such as the study conducted by Ref. [26] have shown that creativity directly and indirectly affects individual performance. These findings have been further substantiated by subsequent research conducted by Ref. [27] which also observed a positive relationship between creativity and individual performance. Thus, it is reasonable to assert that individual creativity has a positive association with task performance, serving as a proxy for individual performance. The results of this study indicated that individual creativity acts as a mediator in the relationship between knowledge hiding and task performance. The hypothesis proposing that knowledge hiding has an indirect effect on individual performance through individual creativity is statistically validated. This implies that individual creativity plays a mediating role in translating the association of knowledge hiding with task performance. The proposition suggesting that individual creativity serves as a mediator for the association of knowledge hiding on task performance is statistically supported.

Empirical evidence suggests that knowledge-hiding not only detrimentally affects task performance [19], but also has a negative impact on creativity (Cerne et al., 2014; Fong et al., 2017; [24]. Contrarily, creativity has been found to positively influence performance [26,27]. The lack of access to essential knowledge due to its concealment or withholding by knowledge-hiding perpetrators can impede the generation of creativity, consequently causing delays in individual performance completion. These findings contribute to the existing body of knowledge overall, by highlighting the importance of individual creativity in enhancing task performance. Additionally, they provide insights into the mediating role of individual creativity in the relationship between knowledge-hiding and task performance. By recognizing the mediating effect of individual creativity, organizations can better understand the mechanisms through which knowledge-hiding associates performance outcomes. Thus, it can hopefully implement strategies to foster and leverage individual creativity to improve task performance.

## 6 Conclussion

Through second-order confirmatory factor analysis, this study tested the indicators and dimensions of knowledge-hiding. The findings revealed that all indicators significantly manifested the dimensions of evasive hiding, playing dumb, and rationalized hiding, thus confirming the construct of knowledge-hiding as defined by Ref. [8]. Among these dimensions, playing dumb was found to be the most prevalent in Indonesia, representing the safest form of knowledge-hiding that avoids equal treatment compared to rationalized hiding and evasive hiding. Furthermore, the results demonstrated a significant negative association of knowledge-hiding on individual task performance. On the contrary, individual creativity exhibited a positive and significant association with individual task performance. These findings highlight knowledge-hiding as a detrimental behavior within organizations, negatively affecting interpersonal relationships and subsequently impacting individual and organizational performance. Moreover, the study revealed that individual creativity serves as a mediator in the relationship between knowledge hiding and task performance. It was observed that individual creativity directly and significantly contributes to task performance. In summary, this research confirmed the presence and significance of knowledge-hiding and its adverse association with individual task performance and creativity. It underscores the importance of fostering individual creativity and addressing knowledge-hiding behaviors to promote positive outcomes in organizational settings.

The findings emphasize the significance of addressing knowledge-hiding behavior and fostering individual creativity to enhance individual task performance within organizations. The research methodology employed a quantitative deductive approach within the positivist paradigm to investigate the mediating role of individual creativity in the relationship between knowledge-hiding and task performance. This study offers significant implications and contributions that enrich our existing knowledge in several crucial ways. Firstly, it adds value by rigorously validating the dimensions and indicators of knowledge hiding through robust statistical analysis. This validation process enhances our comprehension of this behavioral phenomenon, reinforcing the credibility of its measurement. Moreover, the study unveils the adverse consequences of knowledge hiding on individual task performance, underscoring the imperative for organizations to address and rectify this behavior. It accentuates the need for cultivating workplace cultures that emphasize openness and knowledge sharing, as these factors have been shown to augment task performance.

Furthermore, this research extends the existing literature by delving into the intricate dynamics between knowledge hiding, task performance, and the mediating role played by individual creativity. This nuanced investigation broadens our theoretical understanding of how these elements interrelate, shedding light on the complex interplay that can significantly impact organizational performance. This study also equips the financial industry in Indonesia with valuable insights into tackling knowledge hiding, optimizing task performance, and fostering a culture of openness and creativity. It also advances the methodological tools for future research in this sector. These implications are of paramount importance in a field as critical and complex as finance, where efficient decision-making and innovation are pivotal to success.

From a methodological perspective, this study also holds substantial implications. It enhances our methodological toolkit by successfully employing advanced statistical techniques, notably second-order confirmatory factor analysis, to improve the precision of measurement. This methodological advancement contributes to the rigor and accuracy of future research in this domain. Additionally, it suggests the consideration of Covariance-Based Structural Equation Modeling (SEM) with larger sample sizes as a promising avenue for further research. This methodological enhancement would fortify the robustness of future investigations, providing deeper insights into the intricate relationships explored in this study. In summary, this study not only enriches our theoretical understanding of knowledge hiding but also underscores its practical implications for organizations striving to optimize task performance. It bolsters our methodological repertoire and lays the groundwork for future investigations that can delve even deeper into this critical area of study.

## 7 Limitations and future study recommendations

This study has several limitations that should be acknowledged. First and foremost, the study's quantitative and cross-sectional design limits the ability to establish cause-and-effect relationships among the variables. To gain a better understanding of how knowledge hiding affects task performance over time, future research should consider using a longitudinal design. Additionally, this study relied exclusively on self-reported data, which can introduce potential biases such as social desirability and common method variance. To improve the credibility of the findings, it would be beneficial to incorporate multiple data sources, such as supervisor evaluations or objective performance metrics. Furthermore, it's important to note that this study focused on a specific industry (finance) and a particular country (Indonesia), which could constrain the generalizability of the results. To gain a more comprehensive understanding of the connections between knowledge hiding, individual creativity, and performance outcomes, it would be valuable to explore a wider range of organizational settings and cultural contexts.

In addition to these considerations, future research should also explore other potential variables that might mediate the relationship between knowledge hiding and task performance, such as organizational culture or team dynamics. Addressing these limitations through the use of longitudinal research designs, the inclusion of diverse data sources, exploration of different contexts, and the consideration of potential moderating factors will contribute to a more nuanced understanding of knowledge hiding and its impact on performance. In conclusion, this study provides valuable insights for organizations aiming to improve task performance and foster a culture of knowledge sharing.

## Data availability statement

Data will be made available on request.

## CRediT authorship contribution statement

**Ika Atma Kurniawanti:** Writing – review & editing, Writing – original draft, Visualization, Validation, Software, Resources, Project administration, Methodology, Investigation, Formal analysis, Data curation, Conceptualization. **Djumilah Zain:** Writing – original draft, Validation, Supervision, Resources, Methodology, Formal analysis, Data curation. **Armanu Thoyib:** Writing – review & editing, Visualization, Validation, Supervision, Formal analysis, Data curation. **Mintarti Rahayu:** Writing – review & editing, Validation, Supervision, Formal analysis, Conceptualization.

## Declaration of competing interest

The authors declare the following financial interests/personal relationships which may be considered as potential competing interests:The authors report administrative support was provided by Doctoral Program of Management Science, Faculty of Economics and Business, Brawijaya University.
