# Supplementary material for: Knowledge hiding and individual task performance: The role of individual creativity as mediator
Source: Heliyon. 2023 Oct 21;9(11):e21035. doi: 10.1016/j.heliyon.2023.e21035 (PMC10632682; doi:10.1016/j.heliyon.2023.e21035)
Supplement: Multimedia component 1 [file mmc1.docx]

| Evasive Hiding | Frequency | | | | | | | Mean |
| --- | --- | --- | --- | --- | --- | --- | --- | --- |
|  | 1 | 2 | 3 | 4 | 5 | 6 | 7 |  |
| I agree to help even if it's out of compulsion | 75 | 78 | 48 | 30 | 16 | 8 | 1 | 2.46 |
| I agree to help but will provide information that does not exactly match the information requested | 109 | 76 | 35 | 22 | 9 | 5 | 0 | 2.07 |
| I told them I would help later, but I would hold off on helping as long as I could | 139 | 83 | 28 | 4 | 2 | 0 | 0 | 1.62 |
| I will try to offer other information, but different from what was requested | 93 | 80 | 37 | 28 | 9 | 5 | 4 | 2.26 |
|  |  |  |  |  |  |  |  |  |
|  |  |  |  |  |  |  |  |  |
| Playing Dumb | Frequency | | | | | | | Mean |
|  | 1 | 2 | 3 | 4 | 5 | 6 | 7 |  |
| I pretended not to know the requested information | 139 | 83 | 22 | 9 | 2 | 1 | 0 | 1.65 |
| I would say I don not know about the information requested, but the fact is I do | 156 | 72 | 21 | 4 | 1 | 1 | 1 | 1.55 |
| I pretended not to know about the information being discussed | 141 | 80 | 23 | 9 | 3 | 0 | 0 | 1.64 |
| I confess that I am not very familiar with the topic or information | 87 | 84 | 38 | 26 | 16 | 5 | 0 | 2.28 |
|  |  |  |  |  |  |  |  |  |
|  |  |  |  |  |  |  |  |  |
| Rationalized Hiding | Frequency | | | | | | | Mean |
|  | 1 | 2 | 3 | 4 | 5 | 6 | 7 |  |
| I explained that I actually wanted to share the information, but I was not allowed to | 70 | 90 | 44 | 31 | 11 | 8 | 2 | 2.43 |
| I explained that the information is confidential and can only be known by people involved in certain projects. | 48 | 92 | 46 | 36 | 15 | 19 | 0 | 2.75 |
| I informed that my supervisor does not allow anyone to share this information | 52 | 93 | 43 | 36 | 20 | 12 | 0 | 2.67 |
| I declare that I will not answer related questions | 92 | 76 | 33 | 37 | 14 | 4 | 0 | 2.29 |
|  |  |  |  |  |  |  |  |  |
|  |  |  |  |  |  |  |  |  |
|  |  |  |  |  |  |  |  |  |
|  |  |  |  |  |  |  |  |  |
| Individual Creativity | Frequency | | | | | | | Mean |
|  | 1 | 2 | 3 | 4 | 5 | 6 | 7 |  |
| I have the opportunity to use my creative skills and abilities at work | 0 | 0 | 1 | 19 | 31 | 99 | 106 | 6.13 |
| I was invited to submit ideas for improvement and development in the workplace | 0 | 0 | 2 | 26 | 54 | 88 | 86 | 5.90 |
| I have the opportunity to participate in a team | 0 | 0 | 0 | 10 | 26 | 101 | 119 | 6.29 |
| I have freedom to decide how I will complete my work tasks | 0 | 2 | 2 | 41 | 61 | 204 | 202 | 6.29 |
| My creative abilities are fully utilized to develop my potential | 0 | 0 | 4 | 26 | 26 | 101 | 99 | 6.04 |
|  |  |  |  |  |  |  |  |  |
|  |  |  |  |  |  |  |  |  |
| Individual Task Performance | Frequency | | | | | | | Mean |
|  | 1 | 2 | 3 | 4 | 5 | 6 | 7 |  |
| Individual employees always plan their work to be completed on time | 0 | 0 | 1 | 4 | 19 | 96 | 136 | 6.41 |
| Individual employees determine the targets or results to be achieved in their work | 0 | 0 | 1 | 10 | 22 | 99 | 124 | 6.31 |
| Individual employees are able to separate the main problems and side problems at work | 0 | 0 | 0 | 6 | 19 | 111 | 120 | 6.35 |
| Individual employees are able to do a good job with optimal time and effort | 0 | 0 | 3 | 15 | 24 | 116 | 98 | 6.14 |
